# Clinical features of COVID-19 among patients with end-stage renal disease on hemodialysis in the context of high vaccination coverage during the omicron surge period: a retrospective cohort study

**DOI:** 10.1186/s12882-023-03219-w

**Published:** 2023-06-27

**Authors:** Nam-Seon Beck, Soomin Song, Taesung Park, So-Hyeon Hong, Jang Jeong-Eun, Kyoung-Hwan Kim, Joung-Il Im, Sae-Yong Hong

**Affiliations:** 1 Department of Pediatrics, Chung-Ang Jeil Hospital, Chungbuk, South Korea; 2 Department of Statistics, Seoul National University, Seoul, South Korea; 3 Division of Endocrinology and Metabolism, Department of Internal Medicine, Ewha Woman’s University, School of Medicine, Seoul, South Korea; 4 Department of Nursing, Chung-Ang Jeil Hospital, Chungbuk, South Korea; 5 Department of Family Medicine, Chung-Ang Jeil Hospital, Chungbuk, South Korea; 6 Department of Orthopedic Surgery, Chung-Ang Jeil Hospital, Chungbuk, South Korea; 7 Department of Nephrology, Chung-angJeil General Hospital, 24 Jungang-Bukro, Jincheon County, Chungbuk, 27832 South Korea

**Keywords:** COVID-19, COVID-19 vaccination, End-stage renal disease, Hemodialysis, Omicron variant

## Abstract

**Background:**

We determined the clinical presentation and outcomes of the Omicron variant of severe acute respiratory syndrome coronavirus 2 infection in hemodialysis patients and identified the risk factors for severe coronavirus disease (COVID-19) and mortality in the context of high vaccination coverage.

**Methods:**

This was a retrospective cohort study involving hemodialysis patients who were vaccinated against COVID-19 during March–September 2022, when the Omicron variant was predominant, and the COVID-19 vaccination rate was high. The proportion of people with severe COVID-19 or mortality was evaluated using univariate logistic regression.

**Results:**

Eighty-three (78.3%) patients had asymptomatic/mild symptoms, 10 (9.4%) had moderate symptoms, and 13 (12.3%) had severe symptoms. Six (5.7%) patients required intensive care admission, two (1.9%) required mechanical ventilation, and one (0.9%) was kept on high-flow nasal cannula. Of the five (4.7%) mortality cases, one was directly attributed to COVID-19 and four to pre-existing comorbidities. Risk factors for both severe COVID-19 and mortality were advanced age; number of comorbidities; cardiovascular diseases; increased levels of aspartate transaminase, lactate dehydrogenase, blood urea nitrogen/creatinine ratio, brain natriuretic peptide, and red cell distribution; and decreased levels of hematocrit and albumin. Moreover, the number of COVID-19 vaccinations wasa protective factor against both severe disease and mortality.

**Conclusions:**

Clinical features of hemodialysis patients during the Omicron surge with high COVID-19 vaccination coverage were significant for low mortality. The risk features for severe COVID-19 or mortality were similar to those in the pre-Omicron period in the context of low vaccination coverage.

## Background

Patients with end-stage renal disease (ESRD)are susceptible to severe coronavirus disease (COVID-19)due to increased age and comorbidities [1, 2]. It is speculated that cytokines and uremic toxins upregulate the inflammatory milieu, leading to immune impairment among patients with COVID-19 with ESRD [3]. Consequently, the mortality rate of these patients is higher than that of the general population [4, 5]. Furthermore, frequent contact with the health care system for hemodialysis results in a higher incidence of COVID‐19 in this group [6, 7]. Population-based studies have indicated an approximately four-fold increase in mortality among patients on dialysis compared with the general population after accounting for confounding factors [8, 9].

Humoral response after COVID-19 vaccination has been reported to be lower in dialysis patients comparedwithhealthy controls [10, 11]. However, the cellular immune response elicited by vaccination could be preserved in patients with ESRD. This might be associated with increased susceptibility to severe acute respiratory syndrome coronavirus2 (SARS-CoV-2) infection [12, 13]. However, hospitalization and mortality in these patients could be prevented by completing their vaccination [14, 15].

The Omicron (B.1.1.529) variant was first isolated in Africa in late 2021, after which it spread globally. The variant has evolved into several subvariants with significant evasion of immunity elicited by vaccination or prior infections [16, 17]. However, T-cell responses induced by vaccines have acceptable cross-reactivity against variants [18, 19] that protect against severe diseases and mortality [20]. It has been reported that symptoms of the Omicron variant in the general population [21] and those on HD [22, 23] were milder and shorter than those of the previous and wild-type variants.

The Omicron variant reached South Korea in early 2022 and became predominant from March to September 2022. During this period, the Korean population experienced high vaccination coverage against COVID-19.In total,87.7% of the population had received primary series vaccination, 86.8% received the first booster dose, and 64.5% the second booster dose [24].

The emergence of the Omicron variant and high vaccination coverage could significantly affect the disease course among patients with ESRD. However, studies on their impacton patients withCOVID-19with ESRDare limited except for a few observations [15, 23]. Some laboratory features predict the clinical course of patients with COVID‐19, [25] although limited data are available for patients with ESRDwho are fully vaccinated against COVID-19.

Therefore, this study aimed to analyze the clinical presentation and outcomes of dialysis patients with Omicron variant infection in the context of high vaccination coverageand identify risk factors for severe COVID-19 and mortality inpatientsundergoinghemodialysis.

## Methods

### Ethical considerations.

This study was approved by the Public Institutional Review Board of the Ministry of Health and Welfare of South Korea (http://irb.or.kr/menu02/summary.aspx, approval no.: P01-202,209–01-020). The study was carried out in accordance with the Declaration of Helsinki. The need for informed consent was waived by the review board due to the retrospective nature of the study.

### Study design and participants

Since the declaration of the pandemic, an active surveillance system has been implemented in South Korea, which works in close collaboration with private healthcare facilities. This system mandates all individuals with COVID-19 symptoms or epidemiological links to undergo COVID-19 testing. Moreover, all asymptomatic individuals at risk for severe COVID-19have free access to COVID-19 testing, including those with ESRD. Once confirmed, patients with ESRDare referred to designated health facilities to receive appropriate medical care and maintenancehemodialysis under isolation. Upon release after clinical recovery and isolation period (10–14 days after symptom onset), the patients are referred back to their dialysis centers to continuemaintenance hemodialysis.

This was a retrospective cohort study conducted at Chung-Ang JeilHospital, a secondary hospital coveringJincheonCountyand the surrounding areas of theChungbukprovince of SouthKorea, with an approximate population of 200,000. The study period ranged from March to September 2022, when the Omicron variant was predominant.

In total, 106 patients with ESRD aged ≥ 18 years that had been referred to Chung-Ang JeilHospital by the Provincial Ministry of Public Health (MOPH) for critical care and hemodialysisunder isolation after confirmation of COVID-19 were included in this study. The diagnosis of COVID-19 was based on nasopharyngeal swab positivity for SARS-CoV-2 by polymerase chain reaction (PCR) or rapid antigen testfor the suspect cases who meet the WHO clinical criteria and/or have epidemiological links [26].

### Data collection

After reviewing the electronic medical records, data for the following variables were collected: age, sex, body mass index (BMI),fever, and comorbidities (diabetes mellitus, hypertension, history of lung diseases, stroke, cancer, coronary artery disease, history of congestive heart failure, affective disorder, and psychosis).Furthermore, radiologic and laboratory findings (lung computed tomography [CT], serum albumin,complete blood counts with red blood cell and platelet indices, liver profiles with bilirubin and transaminase, lactate dehydrogenase [LDH], brain natriuretic peptide [BNP], D-dimer,and inflammatory markers, includingC-reactive protein [CRP], presepsin, and procalcitonin) were also collected.

Clinical severity of COVID-19was determined based on theWHO criteria as follows [27]:mild, symptomatic patients meeting the case definition of COVID-19 without any evidence of lung infiltration or hypoxia; moderate individuals with clinical (fever, cough, dyspnea, fast breathing) or radiologic signs of pneumonia but no signs of abnormal oxygen saturation (SpO_2_ < 90%) in room air; severe, individuals with clinical signs of pneumonia (fever, cough, dyspnea) plus one of the following: respiratory rate > 30 breaths/min, severe respiratory distress, or SpO_2_ < 90% in room air; critical, individuals with acute respiratory distress syndrome (ARDS).

For radiologic scoring of lung CT images, the lung was divided into five lobes or segments according to the anatomical structures. The pathologic involvement of each lobe was estimated as: no lesion, 0; < 5%, 1; < 25%, 2; ≥ 25% but < 50%, 3; ≥ 50% but < 75%, 4; and ≥ 75%, 5. Semi-quantitative scoring between 0 and 25 was performed for each case [28].

### Statistical analyses

Statistical analyses were performed using R Statistical Software, version 4.1.2. Categorical variables are described as count and frequency, while numeric variables are described as the mean ± standard deviation. Univariate logistic regression was used to explore the association of clinical characteristics and laboratory parameters with oxygen requirements (severe COVID-19) and mortality. Odds ratios (ORs) and 95% confidence intervals (CIs) were calculated for all regression analyses. We applied the “adjusted Woolf” method to zero count samples to estimate small sample CIsfor OR [29]. Statistical significance was defined as a two-sided p-value of < 0.05.

## Results

Demographic and clinical profiles of the patients are presented in Table 1. In total, 106 patients were included in this analysis. The mean age of the study population was 65.6 ± 12.0 years, and 47.2% were male individuals. None of the patients had a history of COVID-19. Comorbidities included hypertension (80.2%), diabetes (79.2%), cardiovascular disease (16.9%), cerebrovascular accidents (13.2%), malignant neoplasm (8.5%), depression or psychosis (6.6%), and severe obesity (BMI > 30 kg/m^2^, 5.7%).

**Table 1:** Patient characteristics on presentation

Patient characteristics	Values
No. of patients	106
Age (years, mean ± SD)	65.6 ± 12.0
Males (N, %)	50(47.2%)
History of prior COVID-19	0 (0%)
Number of vaccinations (N, %)	
0	20(18.9%)
1	0(0%)
2	7(6.6%)
3	73(68.9%)
4	6(5.7%)
Comorbidities (N, %)	
Hypertension	85(80.2%)
Diabetes	84(79.2%)
Cardiovascular disease	18(16.9%)
Cerebrovascular accidents	14(13.2%)
Malignant neoplasm	9(8.5%)
Depression or psychosis	7(6.6%)
Severe obesity (BMI > 30)	6(5.7%)
Duration of symptoms prior to admission (days)	1.31 ± 0.92
Disease severity (N, %)	
Asymptomatic/mild	83(78.3%)
Moderate	10(9.4%)
Severe	13(12.3%)

Categorical variables are described as counts (N) and frequencies(%), while numeric ones are described as means ± standard deviation (SD)

*BMI* body mass index

Eighty-six patients had received vaccination against COVID-19.Thisincluded two shots for seven (6.6%) individuals, three shots for 73 (68.9%), and four shots for six (5.7%); 20 (18.9%) patients did not receive any shots. This indicates that 81.1% of the study population had received at least two vaccine doses.

Regarding the clinical features atthetime of referral, 83(78.3%) patients were either asymptomatic or presented with mild symptoms, 10 (9.4%) had pneumonia but normal oxygen saturation (moderateCOVID-19), while 13 (12.3%) required oxygen supply to maintain SpO_2_ > 93% (severeCOVID-19). No patient presented with ARDS (critical COVID-19) on referral. Four (3.8%) patients who were asymptomatic or had mild symptoms at the time of presentation eventually progressed to severe COVID-19, requiring oxygen supplementation.

Regarding clinical care during hospitalization, 52.8% (56of 106) of those hospitalized received low molecular weight heparin. Six (5.7%) patients required intensive care admission, while two (1.9%) required mechanical ventilation, and one (0.9%) was kept on high-flow nasal cannula.AmongpatientswithsevereCOVID-19, 88.2% (15 of 17) were administered glucocorticoids.

During hospitalization and up to 1 month after discharge, five (4.7%) patients died. However, only one mortality was directly attributed to COVID-19(ARDS), while three were caused by myocardial infarction and the other by heart failure.

**Fig. 1:**
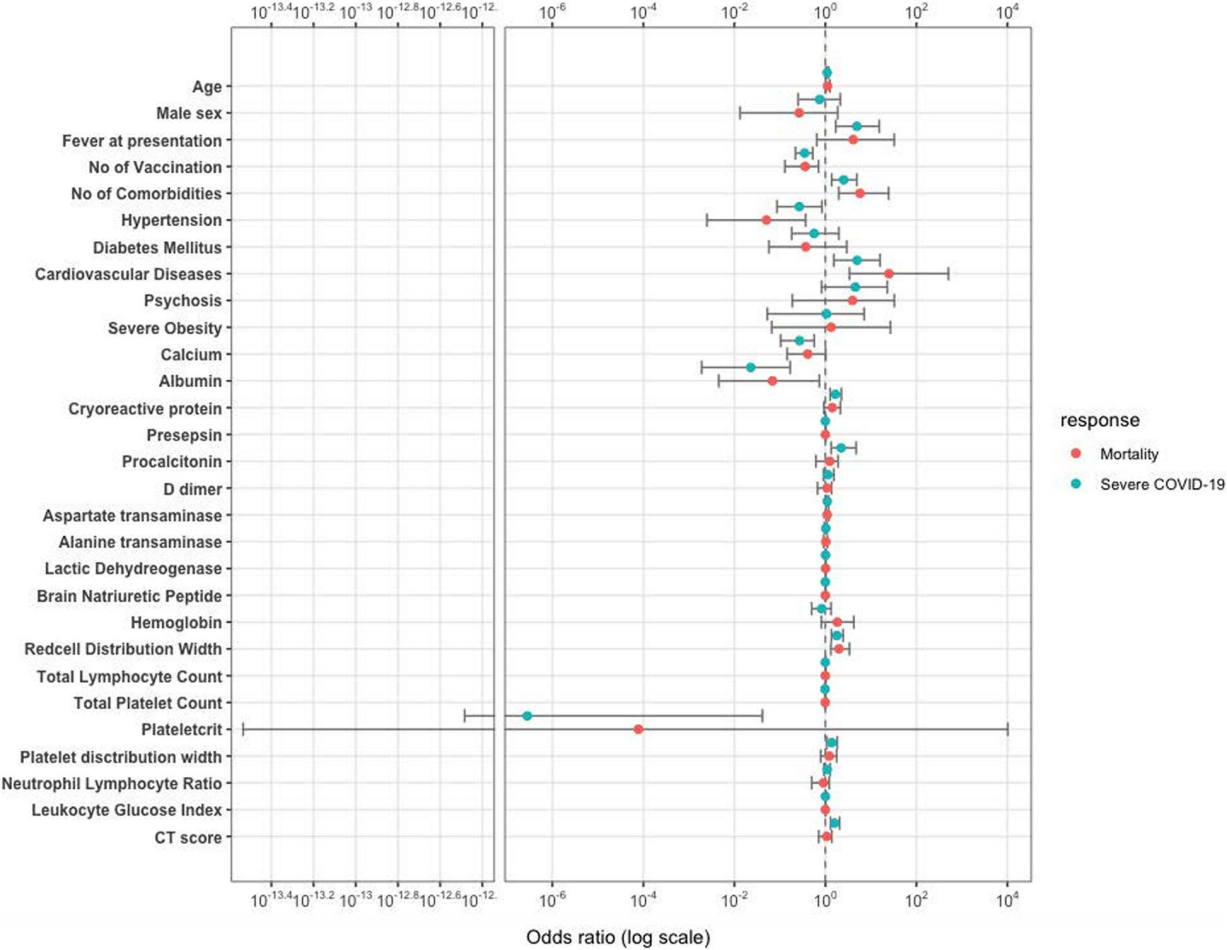
Odds ratio for each clinical and laboratory feature against oxygen demand or mortality. The95% confidence interval for odds ratio (OR) for each clinical and laboratory parameter is shown. The following parameters were identified as significant (*p* < 0.05) for severe COVID-19 (blue dot) by univariate logistic regression analysis: advanced age, fever at presentation, number of comorbidities, pre-existing cardiovascular disease, decreased albumin level, increased levels of aspartate aminotransferase (AST), lactatedehydrogenase (LDH), and lung CT score, increased red cell distribution width (RDW), thrombocytopenia, increased plateletcrit, platelet distribution width (PDW), hypocalcemia and increased BNP and acute phase reactants(inflammatory markers) such as CRP, presepsin, and procalcitonin. Those significant for mortality (red dot) include advanced age, number of comorbidities, pre-existing cardiovascular disease, hypoalbuminemia, increased AST, LDH, RDW, and BNP. Meanwhile, number of vaccination was protective both for severe COVID-19 and mortality. Severe obesity (body mass index > 30 kg/m^2^). Leukocyte glucose index is defined as the product between blood leukocyte counts and glucose levels divided by 1,000. CT score, lung computed tomography score

Univariate logistic regression analysis revealed the following risk factors for severe COVID-19: advanced age; fever at the time of presentation; number of comorbidities; pre-existing cardiovascular disease; decreased albumin level; increased levels ofaspartate aminotransferase (AST), LDH, and lung CT score; increased red cell distribution width (RDW); thrombocytopenia, increasedplateletcrit, and platelet distribution width;hypocalcemia;and increased BNP and acute phase reactants(inflammatory markers) such as CRP, presepsin, and procalcitonin. The variables identified as significant for mortality included advanced age; number of comorbidities; pre-existing cardiovascular disease;hypoalbuminemia;and increasedlevelsofAST, LDH, RDW, and BNP. Meanwhile, hypertension and number of vaccinations were found to be protective factors against both severe COVID-19 and mortality (Fig. 1, Tables 2 and 3).

**Table 2:** Univariate logistic regression analysis of clinical and laboratory features against oxygen demand (severe disease)

**Variables**	**Not severe COVID-19** ***N* = 89**	**Severe COVID-19** ***N* = 17**	**p**	**OR**^a^** (95% CI)**
Clinical features				
Age (years, mean ± SD)	**64.1 ± 11.8**	**73.8 ± 9.8**	**0.004**	**1.09 (1.03, 1.16)**
Male sex (N, %)	43 (48.3%)	7 (41.2%)	0.600	0.75 (0.25, 2.13)
Fever at presentation (N, %)	**20 (22.5%)**	**10 (58.8%)**	**0.004**	**4.93 (1.68, 15.2)**
No. of vaccinations (mean ± SD)	**2.7 ± 0.9**	**0.8 ± 1.4**	** < 0.001**	**0.35 (0.22, 0.53)**
No. of comorbidities (mean ± SD)	**1.9 ± 1.00**	**1.2 ± 0.8**	**0.004**	**2.52 (1.37, 4.90)**
Hypertension (N, %)^b^	**75(84.3%)**	**10 (58.8%)**	**0.021**	**0.27 (0.09, 0.84)**
Diabetes mellitus (N, %)	72(80.90%)	12 (70.6%)	0.300	0.57 (0.18, 1.97)
Cardiovascular diseases (N, %)	**11(12.4%)**	**7 (41.8%)**	**0.007**	**4.96 (1.53, 15.9)**
Psychosis (N, %)	4 (4.5%)	3 (17.7%)	0.063	4.55 (0.83, 22.9)
Severe obesity^c^ (N, %)	5 (5.6%)	1 (5.9%)	> 0.9	1.05 (0.05, 7.11)
Laboratory features (mean ± SD)				
Calcium (mg/dL)	**9.1 ± 0.9**	**8.1 ± 1.0**	**0.002**	**1.36 (1.13, 1.74)**
Albumin (mg/dL)	**3.4 ± 0.3**	**3.1 ± 0.4**	** < 0.001**	**0.02 (0.00, 0.17)**
C-reactive protein (mg/dL)	**1.7 ± 1.6**	**3.7 ± 2.1**	** < 0.001**	**1.66 (1.27, 2.25)**
Presepsin (pg/mL)	**3,881 ± 3,550**	**2,491 ± 1,927**	**0.041**	**1.00 (1.00, 1.00)**
Procalcitonin (μg/L)	**0.7 ± 0.8**	**1.9 ± 2.1**	**0.013**	**2.22 (1.34, 4.74)**
D-dimer (μg/mL)	1.2 ± 2.2	2.0 ± 1.1	0.300	1.13 (0.91, 1.54)
Aspartate transaminase (μ/L)	**18.6 ± 7.7**	**32.5 ± 21.1**	**0.002**	**1.10 (1.04, 1.17)**
Alanine transaminase (μ/L)	18.0 ± 8.3	19.5 ± 8.1	0.500	1.02 (0.958, 1.08)
Lactatedehydrogenase (u/l)	**388.0 ± 91.4**	**475.2 ± 207.2**	**0.002**	**1.01 (1.00, 1.01)**
Brain natriuretic peptide (pg/mL)	**10,974 ± 11,174**	**24,823 ± 14,452**	**0.001**	**1.00 (1.00, 1.00)**
Hemoglobin (mg/dL)	10.5 ± 1.0	10.2 ± 1.4	0.400	0.83 (0.50, 1.33)
Red cell distribution width (%)	**14.1 ± 1.6**	**16.5 ± 2.7**	** < 0.001**	**1.78 (1.36, 2.45)**
Total lymphocyte count (/mm^3^)	1,188.5 ± 542.8	916.2 ± 274.5	**0.051**	1.00 (0.997, 1)
Total platelet count (10^3^/mm^3^)	**168.3 ± 55.8**	**131.7 ± 35.9**	**0.013**	**0.98 (0.97, 1.00)**
Plateletcrit (%)	**0.2 ± 0.1**	**0.1 ± 0.0**	**0.020**	**0.00(0.00,0.041)**
Platelet distribution width (%)	**10.5 ± 1.8**	**11.9 ± 2.3**	**0.014**	**1.38 (1.08, 1.82)**
Neutrophil lymphocyte ratio	5.4 ± 3.1	6.6 ± 2.6	0.200	1.09 (0.93, 1.27)
Leukocyte glucose index^d^	901.7 ± 536.9	999.3 ± 1,012.9	0.600	1.00 (0.999, 1)
CT score^e^(mean ± SD)	**0.5 ± 1.7**	**4.2 ± 3.6**	** < 0.001**	**1.59 (1.29, 2.05)**

Categorical variables are described as counts (N) and frequencies (%), while numeric variables are described as means ± standard deviation (SD)

^a^OR: odds ratio

^b^Hypertension: refer to the discussion^2^

^c^Severe obesity is defined as body mass index > 30 kg/m^2^

^d^Leukocyte glucose index is defined as the product between blood leukocyte counts and glucose levels divided by 1000

^e^CT score: lung computed tomography score

^*^Source: own calculation

The figures in bold represent statistical significance *p*<0.05

**Table 3:** Univariate logistic regression analysis of clinical and laboratory features against mortality

Variables	Alive *N* = 101	Died *N* = 5	p	OR (95% CI)^a^
Clinical features				
Age (years, mean ± SD)	**65.1 ± 12.0**	**77.0 ± 4.8**	**0.034**	**1.12 (1.02, 1.26)**
Male sex (N, %)	49 (48.5%)	1 (20.0%)	0.200	0.27 (0.013, 1.87)
Fever at presentation (N, %)	27(26.7%)	3 (60.0%)	0.130	4.11 (0.65, 32.5)
No. of vaccinations (mean ± SD)	**2.5 ± 1.2**	**0.6 ± 1.2**	**0.009**	**0.36 (0.13, 0.71)**
No. of comorbidities (mean ± SD)	**1.2 ± 0.8**	**2.6 ± 0.8**	**0.004**	**5.75 (1.97, 24.4)**
Hypertension^c^ (N, %)	**84 (83.2%)**	**1 (20.0%)**	**0.009**	**0.05(0.00, 0.37)**
Diabetes mellitus (N, %)	81 (80.2%)	3 (60.0%)	0.300	0.37 (0.06, 2.95)
Cardiovascular diseases (N, %)	**14 (13.9%)**	**4 (80.0%)**	**0.005**	**24.9 (3.38, 50.6)**
Psychosis (N, %)	6 (5.9%)	1 (20%)	0.200	3.96 (0.187, 32.7)
Severe obesity^b^ (N, %)	6 (5.9%)	0 (0%)	0.85^f^	1.34(6.64, 26.9)^f^
Laboratory features (mean ± SD)				
Calcium (mg/dL)	9.0 ± 1.0	8.1 ± 0.6	0.058	0.41 (0.14, 1.01)
Albumin (mg/dL)	**3.4 ± 0.3**	**3.0 ± 0.2**	**0.028**	**0.07 (0.00, 0.737)**
C-reactive protein (mg/dL)	2.0 ± 1.8	3.5 ± 1.9	0.088	1.42 (0.93, 2.14)
Presepsin (pg/mL)	2,633 ± 2,211	4,492 ± 3,356	0.110	1.00 (1.00, 1.00)
Procalcitonin (μg/L)	0.8 ± 1.2	1.3 ± 0.8	0.400	1.25 (0.62, 1.9)
D dimer (μg/mL)	1.3 ± 2.1	1.9 ± 0.9	0.500	1.09 (0.67, 1.37)
Aspartate transaminase (μ/L)	**19.5 ± 8.3**	**47.4 ± 29.9**	**0.005**	**1.09 (1.04, 1.19)**
Alanine transaminase (μ/L)	18.1 ± 8.3	20.0 ± 7.8	0.600	1.02 (0.91, 1.12)
Lactatedehydrogenase (μ/L)	346.7 ± 95.4	633.6 ± 278.5	**0.006**	1.01 (1.00, 1.02)
Brain natriuretic Peptide (pg/ml)	**12,161 ± 11,967**	**28,706 ± 13,596**	**0.019**	**1.00 (1.00, 1.00)**
Hemoglobin (mg/dl)	10.4 ± 1.1	11.1 ± 1.1	0.140	1.83 (0.82, 4.22)
Red cell distribution width (%)	**14.3 ± 1.8**	**17.7 ± 2.7**	**0.003**	**1.99 (1.32, 3.38)**
Total lymphocyte count (/mm^3^)	1,151.2 ± 523.3	1041.5 ± 327.5	0.700	1.00 (1.00, 1.00)
Total platelet count (10^3^/mm^3^)	163.4 ± 54.8	143.2 ± 47.7	0.400	1.00 (097, 1.01)
Plateletcrit (%)	0.2 ± 0.1	0.1 ± 0.1	0.400	0.00 (0.00,10,200)
Platelet distribution width (%)	10.7 ± 2.0	11.6 ± 1.1	0.300	1.22 (0.08, 1.77)
Neutrophil lymphocyte ratio	5.6 ± 3.1	5.1 ± 1.4	0.700	0.91 (0.50, 1.20)
Leukocyte glucose index^d^	931.5 ± 641.2	631.8 ± 272.6	0.300	1.00 (1.00, 1.00)
CT score^e^(mean ± SD)	1.1 ± 2.6	1.6 ± 1.5	0.600	1.07 (0.713, 1.38)

Categorical variables are described as counts (N) and frequencies (%), while numeric variables are described as means ± standard deviation (SD)

^a^OR: odds ratio

^b^Severe obesity: body mass index > 30.3 kg/m^2^

^c^Hypertension: refer to the discussion

^d^Leukocyte glucose index is defined as the product between blood leukocyte counts and glucose levels divided by 1000

^e^CT score: lung computed tomography score

The figures in bold represent statistical significance *p*<0.05

^f^Source: own calculation

## Discussion

In total, five mortality cases were observed during the study period, with three attributed to myocardial infarction, one to heart failure, and one to ARDS. This implies that most deaths were not directly attributable to COVID-19. This result is strengthened by the observation that lungCT score was not a significant predictor of mortality but of severe disease by univariate logistic regression. This finding is not in concordance with aprevious study in whichthelung CT score was considered a significant predictor of mortality [30].

Notably, vaccination was a significant protective factor againstsevere disease and mortality due to comorbidities during and up toonemonth after active SARS-CoV-2 infection. It might also indicate that vaccination and/or lower viral virulence could reduce mortality directly attributable to COVID-19 but not mortality caused by comorbidities. At the same time, the result could also support the previous observation that patients with ESRDcould still be at risk of dying from othercauses, even after recovery from COVID-19 [31].

Similar to observation among patients withCOVID-19 on HD [22], the observed mortality rate of 4.7% (5 of 106) among patients with COVID-19 with ESRD in our study was comparatively lower than that among those with ESRDbeforethe Omicron era (22.4%; February 2020 to November 2021) in South Korea [32]. This pre-Omicron mortality rate is comparable to other reported series among patients with ESRD,which demonstrated a mortality rate of approximately 20–30% [33].

Despite the high proportion of asymptomatic and mild cases in our study (78.3%), the mortality rate is still considered lower thanthatof a study thatshowed similar disease severity (79% with either asymptomatic or mild cases) but higher mortality (18%) [34].

Among the general population of South Korea, theCOVID-19 mortality rate during the Omicron period was relatively low (0.13%) [35]. This favorable outcome may be explained by several factors, including high vaccination coverage against COVID-19, the Omicron subvariants, an efficient healthcare system, and active cooperation between the private sector and central government [24]. The virulence of the Omicron subvariants may be reduced due to the unceasing development in preventive and therapeutic measures duringthepandemic [33] and cumulative acquired immunity by natural infections. However, considering the lack of differences in baseline characteristics, clinical care, negligible cumulative non-Omicron cases resulting in acquired immunity, and public health policies for the ESRDpopulation between this study and that of the pre-Omicron period in South Korea, [32] the low mortality (4.7% vs. 22.4%) could be attributed to the lower virulence of the variants and/or the protective effect of COVID-19 vaccination. The mid-interval vaccination rate for at least two shots in the South Korean population in the previous study over the pre-Omicron period was 8% (0.02–79%), which is lower than that for this study during the Omicron period, 86% (85–86%) (https://ourworldindata.org/covid-vaccinations?country=~KOR, 2022).

Similar to the previous studies, older patients tended to show a poorer prognosis [8, 36, 37]. Comorbidities such as hypertension, diabetes mellitus, and cardiovascular diseases were also found to be risk factors for severe forms of the disease [38–40].

In this study, nearly all patients (96.2%) had at least one underlying disease, including hypertensionin80.1%, diabetes in79.2%, cardiovascular diseases in 17.0%, and cerebral vascular accidents in 13.2% of the patients.

Contrary to our expectations, high blood pressure was identified as a “protective” factor for both mortality and severe COVID-19. Low systolic blood pressure(SBP < 125 mmHg) was an indicator of cardiovascular mortality in a large population-based study on older patients with a history of acute myocardial infarction [41]. This could be further precipitated by direct cardiovascular insults caused by COVID-19 [42].

In our study, after the exclusion of four cardiovascular mortality cases presenting with low SBP (< 125 mmHg) and underlying cardiac disease, the proportion of hypertension was not different between the severe and milder (mild or asymptomatic) COVID-19 groups (84.3%, n = 13 vs. 76.9%, n = 89; p = 0.51). Therefore, we speculate that the result might be attributed to cardiogenic hypoperfusion among the mortality cases with cardiovascular disease, not resulting from the true protective effect of hypertension.

Similar to other studies, we observed that some laboratory variables predicted the clinical course of COVID‐19, including increasedlevelsof AST, LDH, and acute phase reactants [25, 43, 44].

Some studies have shown that augmented inflammatory responses with cytokine release syndrome (CRS)are the major contributors to poor clinical outcomes of COVID-19 [45, 46]. Elevated levels of acute phase reactants, LDH, AST, and other hematologic parameters, are important biomarkers of CRS [47]. In the current study, other laboratory parameters such as hypoalbuminemia and increased RDW predicted poor prognosis for disease severity and mortality. Considering four mortality cases of cardiac origin, it was not surprising that BNP was identified as a risk predictor for COVID-19 mortality. It has been reported that BNP value may help identify patients with worse prognoses among those with COVID-19, regardless of troponin levels [48].

This study also identified BNP as a separate risk predictor for severe COVID-19.

Notably, changes in platelet count and platelet indices could also serve as risk factors for severe disease. It is known that cytokines released during systemic inflammation, such as IL-1, IL-6, and TNF-α, play a role in thrombopoiesis. Thus, platelet count and its indices, such asplateletcrit, meanplateletvolume, and plateletdistributionwidth, can be used as markers of inflammation [49].

Limitations of this study includedits small sample size and retrospective design.Because of the small sample size resulting in the issue of perfect separation in the logistic regression model, the outcome of the multivariate analysis was not included in the study. Meanwhile, enrollment of all confirmed patients with COVID-19, regardless of their symptoms and disease severity, was one of our study strengths. However, considering that all referrals were exclusively coordinated by the provincial MOPHand not solely based on scientific evidence, some extent of selection bias could have been involved.

## Conclusions

In conclusion, clinical features of patients with ESRD during the Omicron surge with high COVID-19 vaccination coverage were significant for low mortality, with most cases being attributable to pre-existing comorbidities. However, the risk predictors for severe COVID-19 or death were similartothosein the pre-Omicron period with low vaccination coverage.

## Data Availability

The datasets used and/or analyzed during the current study are available from the corresponding author on reasonable request.

## Abbreviations

ARDS: Acute respiratory distress syndrome
AST: Aspartate aminotransferase
BMI: Body mass index
BNP: Brain natriuretic peptide
CI: Confidence interval
COVID-19: Coronavirus disease
CRP: C-reactive protein
CRS: Cytokine release syndrome
CT: Computed tomography
ESRD: End-stage renal disease
LDH: Lactate dehydrogenase
MOPH: Ministry of public health
PCR: Polymerase chain reaction
RDW: Red cell distribution width
SARS-CoV-2: Severe acute respiratory syndrome coronavirus 2
SBP: Systolic blood pressure
OR: Odds ratio
